# Kinetics of Autoxidation of an Oil Extract from *Terminalia catappa*

**DOI:** 10.4103/0250-474X.41472

**Published:** 2008

**Authors:** E. O. Omeje, G. B. Okide, C. O. Esimone, U. Ajali

**Affiliations:** Department of Pharmaceutical and Medicinal Chemistry, Faculty of Pharmaceutical Sciences, University of Nigeria, Nsukka-410001, Nsukka; 1 Department of Pharmaceutics, Faculty of Pharmaceutical Sciences, University of Nigeria, Nsukka-410001, Nsukka

**Keywords:** Kinetic study, autoxidation, oil extract, *Terminalia catappa*, Tropical almond

## Abstract

Soxhlet extractor was used in the extraction of oil from milled seeds of *Terminalia catappa* using petroleum ether (40-60°). The optimal oil yield was 56.71±1.66% with a viscosity of 40.79±1.05 centipoises. Other parameters of the oil were found as follows; specific gravity-0.9248, refractive index-1.4646, acid value-3.35, peroxide value-8.6, saponification value-166.2, and unsaponifiable matter-1.46. The crude oil extract was water-degummed, bleached and deodorized to generate what we called refined oil. Autoxidation of the crude and refined *T. catappa* oil extract was done at five different temperatures of 0±0.1°, 20±0.1°, 40±0.1°, 60±0.1° and 80±0.1° and also in the presence of pure α-tocopherol at a concentration of 1.0% (w/v) by measuring peroxide value variations over 96 h. In all evaluations, the refined oil exhibited lower tendency towards autoxidation but not at temperatures above 60±0.1°. The use of Arrhenius equation revealed generally very low activation energies of 0.0261 cal/deg×mol and 0.0122cal/deg×mol for crude oil and antioxidant-treated crude oil, respectively and 0.0690 cal/deg×mol and 0.0177 cal/deg×mol for the refined oil. This study indicates *T. catappa* seed oil to be potential pharmaceutical oil with excellent characteristics.

The slow atmospheric oxidation (without combustion) of a C-H bond to a COOH group is called autoxidation 1. It actually applies to any slow oxidation with atmospheric (molecular) oxygen and occurs mostly by free radical pathways 2,3. Generally, the process leads to deterioration inrubbers, plastics, foods, drugs^4^, paints, lubrication oils and so on^5^. Autoxidation is catalyzed by light and catalysts, notably the oxides of heavy metals and various peroxides^6^. Despite the various applications of autoxidation reactions in the oil industry, it remains highly undesirable in pharmaceutical oils and preparations containing them. Kinetics of autoxidation mechanisms in fixed oils have been studied by several workers. Okide and Adikwu recently reported the autoxidation of arachis oil^6^. They established that the reaction completely obeyed a second order kinetic model. The high oil yield from *T. catappa* seed motivated us into the present research. This work is a preliminary study on the kinetics of the autoxidation of *T. catappa* oil and hence will aim to establish the stability and its suitability as potential pharmaceutical oil. We are currently unaware of any such report. *T. catappa,* referred to as Indian almond is commonly grown in the tropics and Asia, where it serves mainly as shades, ornamentand food forman. The fruits of *T. catappa* were harvested from Nsukka, Enugu State of Eastern Nigeria in May 2000; were sun dried and cracked. The nuts were stored in airtight containers and kept away from moisture and light. Later, the seeds were dried in an oven pre-set at 60° for 5 d, milled, sieved with 20 mm sieve and stored at 4° prior to use. Petroleumether (40-60°), chloroform, methanol, methylethylketone, potassium iodide, sodium thiosulphate were sourced commercially and were products of Merck, England. Other reagents were standard laboratory reagents.

Ten grams of the dried ground kernel was extracted with petroleum ether (40-60°) for a period of 16h. using Soxhlet extractor. The solvent was then evaporated *in vacuo* and the residual oil was dried in an oven at 40° to constant weight. A total of 2 kg of milled seed was extracted in 400 g portions each for an optimum period of 3 h. The recovered oil was stored in a well-closed amber-coloured bottle at room temperature. The physicochemical properties of the oil were determined as described in British Pharmacopoeia^7^. The crude oil extract was refined following a modification of the method proposed by Haraldsson^8^. Subsequently, this refined portion of the extract was labeled refinedoil while the untreated portion was referred to as the crude oil throughout this research. Phytochemical analysis was carried out for the presence of alkaloids, tannins, saponnins, glycosides, flavonoids, resins and cyanogenic glycosides in the seed^9^. Autoxidation study was done following the method described by Okide and Adikwu^6^. Starch solution, 0.1 M sodium thiosulphate solution, 2 M tetraoxosulphate VI acid solution, 0.1 M sodium hydoxide solution, 0.1 M oxalic acid solution, 0.1 M potassium iodate solution, were prepared accordingly. The 0.1 M sodium thiosulphate solution was standardized by iodometric titrations. For the autoxidation studies, titrimetric method was employed specifically, iodimetric method. The specific quantities of the crude and refined oil were determined pycnometrically at various temperatures (0±0.1°, 20±0.1°, 40±0.1°, 60±0.1° and 80±0.1°). A total of four different samples of the oil were prepared each for the crude and refined then placed in water bath and agitated mechanically. Two samples contained α-tocopherol at a concentration of 0.1% (w/v) for both crude and refined oil. Oxygen was supplied through reaction vessels with the use of an aerator at constant pressure. At pre-determined intervals, and for a total period of 96 h, 5 ml of the test sample was withdrawn and allowed to drain into a clean dry 250 ml iodine flask. The oil was dissolved with 20 ml of acetic acid and chloroform (3:2) and 1 ml of saturated iodide solution was added. The solution was placed in the dark for 10 min. Exactly 50 ml volume of distilled water was added and titrated with 0.1 M sodium thiosulphate using starch mucilage as indicator. Consequently, peroxide value (PV) was calculated from the following relationship, PV = Volume o fMol/l sodium thiosulphate × 1000/weight of sample. 1. In all calculations for both crude and refined oil, the normality of the thiosulphate was corrected to account for dilutions. This process was repeated at different temperatures using the different samples for both crude and refined oil, and the results recorded.

The proximate composition of the *T. catappa* seed is shown in Table 1 with oil yield of approximately 57%. This implies that the seed is a cheap source of oil that could be of great applications in Pharmacy and in other industries. The comparative physicochemical properties of both the crude and refined *T. catappa* oil are as shown in Table 2. The peroxide value, which is a function of unsaturation, time and type of storage fairly, increased during the refining process with values of 8.59±0.06 to 8.63±0.03 in the crude and refined oil, respectively. This is not inagreement with our expectations but it is an indication that autoxidation could have been initiated by the refining processes. Hence, it is not needful to refine the oil from *T. catappa* seed since the refining did not show any advantage for the purpose of autoxidation studies. In summary, there was no statistically significant (*P*<0.05) difference in the peroxide values of the crude and the refined oil, an indication that refining processes did not greatly affect peroxide value. Autoxidation process in the oil was established by variations inperoxide values over 96 h. Unexpectedly, the already established antioxid antactivity of α-tocopherol was not evident, as the peroxide values of the oil tended to increase with its presence. This aberrant behaviour was also manifested in our effort to confirm that the autoxidation process obeyed second order kinetic model as reported for arachis oil^6^. The generated half-life (T_1/2_) values of 172.00, 152.00, 86.50, 2.71, 5.40 h and 192.00, 76.75, 30.20, 3.07, 2.15 h for crude and refined oil respectively at temperatures between 0 to 80° were suggestive of a second order kinetics especially for the refined since the T_1/2_ declined with increasing temperatures. The low values of activation energies for this autoxidation were 0.0261 and 0.0122 cal/deg×mol for the crude oil in the absence and presence of antioxidants respectively. The refined oil exhibited activation energies of 0.0690 and 0.0177 cal/deg×mol_._ The reaction is very slow, an indication that the re may be an *in situ* antioxidant in the oil.

**TABLE 1:** PROXIMATE COMPOSITION OF *T. CATAPPA* SEED

Constituents	Percentage composition±SEM
Oil	56.71±1.66
Protein	26.30±0.14
Carbohydrate	6.50±0.38
Fibre	4.40±0.01
Ash	4.55±0.45
Moisture	1.54±0.29

SEM is the standard error of mean

**TABLE 2:** PHYSICOCHEMICAL PROPERTIES OF THE *T. CATAPPA* SEED OIL

Analysis/determination	Values±SEM	
	
	Crude oil	Refined oil
Physical state at room temperature	Liquid	Liquid
Colour	Golden	Golden
	yellow	yellow
Odour	Pleasant	Pleasant
Specific gravity	0.9521±0.08	0.9200±0.10
Density	0.9400±0.02	0.9120±0.03
Refractive index	1.4646±0.04	0.4640±0.06
Viscosity (cps)	40.79±1.05	36.80±0.94
Acid value	3.5343±0.14	3.2500±0.11
Peroxide value	8.59±0.06	8.63±0.03*
Saponification value	166.2±3.69	157.8±2.88
Unsaponifiable matter (%)	1.48±0.002	1.02±0.001
Iodine value	38.59±1.14	38.63±1.06

* fair increase in Peroxide value during refining process and no signifocant difference between crude and refined (P<0.05) during autoxidation at different temperatures
